# The relationship between regional inequalities in the provision of emergency health services and other health services

**DOI:** 10.1097/MD.0000000000035930

**Published:** 2023-11-10

**Authors:** Erkan Boğa

**Affiliations:** a Republic of Türkiye Ministry of Health Esenyurt Necmettin Kadioğlu Hospital, Istanbul, Türkiye.

**Keywords:** emergency, inequality, price level index, statistical regions

## Abstract

In this research, it was aimed to examine relationship between regional inequalities in the provision of emergency health services and other health services in Turkey. The values of the number of emergency services and the population per emergency service for the years 2002-2021 were taken from the most up-to-date database published by the Ministry of Health in 2022 and were chosen as the dependent variables of the study. The “regional price level indices for consumption expenditures (PLI)” and “gross domestic product per capita, Statistical Regions Level 2 (PcGDP)” data compiled by TURKSTAT were used as independent variables. Number of emergency stations were significantly correlated with TR31 (İzmir) (*r* = 0.903; *P* < .01), TR32 (Aydin, Denizli, Muğla) (*r* = 0.771; *P* < .01), TR42 (Kocaeli, Sakarya, Düzce, Bolu, Yalova) (*r* = −0.798; *P* < .01), TR62 (Adana, Mersin) (*r* = 0.837; *P* < .01), TR63 (Hatay, K.Maraş, Osmaniye) (*r* = −0.749; *P* < .01), TR72 (Kayseri, Sivas, Yozgat) (*r* = −0.719; *P* < .01), TR83 (Samsun, Tokat, Çorum, Amasya) (*r* = 0.873; *P* < .01), TRA2 (Ağri, Kars, Iğdir, Ardahan) (*r* = −0.873; *P* < .01), TRB2 (Van, Muş, Bitlis, Hakkari) (*r* = −0.736; *P* < .01), TRC2 (Şanliurfa, Diyarbakir) (*r* = 0.697; *P* < .01), and TRC3 (Mardin, Batman, Şirnak, Siirt) (*r* = 0.574; *P* < .01). In total, 11 of 26 were significantly correlated with inequalities. Although the number of emergency services has increased since 2002 and the population density per emergency room has tended to decrease, regional inequalities also have an impact on the delivery of emergency services today.

## 1. Introduction

Emergency services are the units that provide health services and respond first and fastest to health-related emergencies. The speed and duration of the service provided in the emergency services are of vital importance. Therefore, long before quality health services, emergency services should be accessible to all individuals and the public.^[1–4]^ Access and distribution to these places are as important as the increase in the number and efficiency of emergency services.^[5–8]^ For this reason, it is vital that emergency services are effectively communicated to all subgroups within the country, from population density to purchasing power, from technological opportunities to other public services.

Regional inequalities have emerged as a result of urbanization and factorization after industrial production, countries have seen urbanization as a modernization and therefore regional inequalities have emerged. As a result of the intense migration to the industrialized cities due to reasons such as raw materials, markets and supply chain in industrial production, important problems began to be experienced in terms of population distribution.^[9–12]^ The inequality or imbalance of these distributions has affected all areas from education to health, from cleaning and environmental processes to social life and has been the subject of many researches. The most important result of these studies is that regional inequalities can be eliminated with a fair process in the distribution of opportunities and opportunities, especially in public administration.^[13–15]^ However, for this, first of all, the effects of regional inequalities and the current situation must be effectively revealed.

Although it is an important issue to prevent discrimination in terms of status and opportunity differences caused by regional inequalities, and in terms of public administration, the most important issue in this field is the emergency services. Access to all health services is important both for the comfort of life of individuals and for public health. However, access to emergency services is more important than other elective or service health services. Although there have been studies on regional inequalities and health services, there have not been enough studies examining the relationship between regional inequalities and emergency health services. Therefore, in this study, it is aimed to examine the relationship between regional inequalities and emergency services.

## 2. Methods

The research was designed in the descriptive scanning model and is a spatial study. In the study, the values of the number of emergency services and the population per emergency service for the years 2002 to 2021 were taken from the most up-to-date database published by the Ministry of Health in 2022 and were chosen as the dependent variables of the study.

### 2.1. Study design

The study design is cohort study, in which a group of statistical unit changes are observed in a period of time.^[16]^

### 2.2. Study setting and participants

Since the research is a population based study, statistical regions of Turkish Statistical Institute (TURKSTAT) were locations and target populations of the study setting. The “regional price level indices for consumption expenditures-PLI” and “gross domestic product per capita, Statistical Regions Level 2-PcGDP” data compiled by TURKSTAT were used as independent variables. Since the PLI data between the years 2008 to 2021 and the PcGDP data between the years 2004 to 2021 are available, they were included in the study. Due to the time difference between the parameters in the data set, “unbalanced” methods were used. Whole sampling method based on time and selection processes were used. Eligibility criteria were defined as being TURKSTAT classifications. PLI: Regional price level indices for consumption expenditures PcGDP: Gross domestic product per capita, Statistical Regions Level 2.

### 2.3. Data collection

Data used in the study were collected from public data source of Health Annual Reports published by Ministry of Health in 2022 for years between 2002 and 2021. Variables were Regional price level indices for consumption expenditures (PLI), Gross domestic product per capita, Statistical Regions Level 2 and (PcGDP).

### 2.4. Ethical considerations

Since data used I the study are anonymous and may be reached by public, neither ethical approval nor institute permission are not applicable.

### 2.5. Data analysis

Parity analysis, change semiotic analysis and Spearman’s rho correlation analysis were used in the analysis of the data. Parity analysis was used to evaluate yearly change in parameters of the research. Semiotic analysis was used to describe changes and their trends. Spearman’s rho correlation was used for relationship between nonparametric variables due to linearization deviations in regression models. All analyzes were performed in SPSS 25.0 for windows program with 95% confidence interval and 0.05 significance level.

### 2.6. Statistical considerations:

There have not been any adjustments for potential confounders, since the data used in the study are provided from a public and adjusted data from Ministry of Health and TURKSTAT.

### 2.7. Limitations

Limitations of the study are relevant with data restrictions provided by Ministry of Health and TURKSTAT. Since the research is a population based research with population data, it was highly generalizability, and validity provided by TURKSTAT.

### 2.8. Reproducibility

Data used in the study are collected from official website of Ministry of Health (https://www.saglik.gov.tr/TR,84930/saglik-istatistikleri-yilliklari.html, Retrieved: 22.06.2023) and TURKSTAT (https://www.tuik.gov.tr/, Retrieved: 20.06.2023), and can be reproduced for further studies.

## 3. Results

Cities and codes according to TURKSTAT 2021 statistically regional classification were given in the Table 1. The number of emergency services, which was 481 in 2002, reached 3170 in 2021. The number of population per emergency department decreased from 138,050 in 2001 to 26,713 in 2021. During this period, while the number of emergency services was increased, the emergency service capacity was also increased. Both the increase in the number of emergency services and the decrease in the number of population per emergency department showed a regular and close to linear distribution within the examined time period (Table 2).

**Table 1:** Cities and codes according to TURKSTAT 2021 statistically regional classification.

Code	Cities
TR10	İstanbul
TR21	Tekirdağ, Edirne, Kirklareli
TR22	Balikesir, Çanakkale
TR31	İzmir
TR32	Aydin, Denizli, Muğla
TR33	Manisa, Afyon, Kütahya, Uşak
TR41	Bursa, Eskişehir, Bilecik
TR42	Kocaeli, Sakarya, Düzce, Bolu, Yalova
TR51	Ankara
TR52	Konya, Karaman
TR61	Antalya, Isparta, Burdur
TR62	Adana, Mersin
TR63	Hatay, K.Maraş, Osmaniye
TR71	Kirikkale, Aksaray, Niğde, Nevşehir, Kirşehir
TR72	Kayseri, Sivas, Yozgat
TR81	Zonguldak, Karabük, Bartin
TR82	Kastamonu, Çankiri, Sinop
TR83	Samsun, Tokat, Çorum, Amasya
TR90	Trabzon, Ordu, Giresun, Rize, Artvin, Gümüşhane
TRA1	Erzurum, Erzincan, Bayburt
TRA2	Ağri, Kars, Iğdir, Ardahan
TRB1	Malatya, Elaziğ, Bingöl, Tunceli
TRB2	Van, Muş, Bitlis, Hakkari
TRC1	Gaziantep, Adiyaman, Kilis
TRC2	Şanliurfa, Diyarbakir
TRC3	Mardin, Batman, Şirnak, Siirt

**Table 2:** Number of emergency services and population per emergency services according to years.

Year	Number of emergency Services	Population per emergency services
2002	481	138.050
2003	494	136.007
2004	890	76.416
2005	976	70.554
2006	1.175	59.345
2007	1.264	55.844
2008	1.308	54.677
2009	1.317	55.096
2010	1.375	53.617
2011	1.710	43.698
2012	1.863	40.594
2013	2.072	37.002
2014	2.186	35.542
2015	2.323	33.896
2016	2.400	33.256
2017	2.618	30.867
2018	2.735	29.983
2019	2.886	28.813
2020	3.050	27.415
2021	3.170	26.713

The region with the highest PLI value between 2008 and 2021 is TR10 Istanbul, followed by TR31 İzmir and TR51 Ankara, respectively. The province group with the lowest PLI value TRC3 is Mardin, Batman, Şirnak, Siirt regions. However, in general, the PLI value was between 114.18 and 93.81 mean values and did not fall below 90% (Table 3).

**Table 3:** Price Level Index for statistical regions for 2008–2021.

	Cities	Minimum	Maximum	Mean	Std. deviation
TR10_PLI	İstanbul	113,09	115,32	114,18	0,6
TR31_PLI	İzmir	105,21	110,39	107,31	1,81
TR51_PLI	Ankara	101,21	107,69	105,38	1,91
TR42_PLI	Kocaeli, Sakarya, Düzce, Bolu, Yalova	102,87	104,82	103,83	0,51
TR90_PLI	Trabzon, Ordu, Giresun, Rize, Artvin, Gümüşhane	102,32	104,43	103,13	0,54
TR21_PLI	Tekirdağ, Edirne, Kirklareli	101,69	103,39	102,67	0,49
TR61_PLI	Antalya, Isparta, Burdur	101,25	104	102,49	0,91
TR41_PLI	Bursa, Eskişehir, Bilecik	100,84	103,72	102,17	1,11
TR22_PLI	Balikesir, Çanakkale	100,55	103,37	102,14	0,91
TR32_PLI	Aydin, Denizli, Muğla	100,58	103,61	101,89	0,99
TR62_PLI	Adana, Mersin	99,07	101,92	100,59	0,94
TR81_PLI	Zonguldak, Karabük, Bartin	98,96	101,47	99,76	0,61
TR33_PLI	Manisa, Afyon, Kütahya, Uşak	97,45	99,31	98,48	0,6
TR83_PLI	Samsun, Tokat, Çorum, Amasya	97,42	99,55	98,36	0,74
TRA1_PLI	Erzurum, Erzincan, Bayburt	97,42	99,33	98,35	0,6
TR82_PLI	Kastamonu, Çankiri, Sinop	97,33	98,21	97,77	0,31
TRB1_PLI	Malatya, Elaziğ, Bingöl, Tunceli	95,13	98,74	97,27	1,02
TR72_PLI	Kayseri, Sivas, Yozgat	96,41	97,68	97,21	0,38
TRC1_PLI	Gaziantep, Adiyaman, Kilis	96,24	98,01	97,07	0,59
TRB2_PLI	Van, Muş, Bitlis, Hakkari	95,19	98,71	97,04	1,07
TR52_PLI	Konya, Karaman	95,73	99	96,96	0,99
TRC2_PLI	Şanliurfa, Diyarbakir	95,55	97,98	96,36	0,65
TR71_PLI	Kirikkale, Aksaray, Niğde, Nevşehir, Kirşehir	94,69	96,55	95,64	0,5
TRA2_PLI	Ağri, Kars, Iğdir, Ardahan	93,34	96,95	95,18	1,36
TR63_PLI	Hatay, K.Maraş, Osmaniye	93,35	96,33	94,96	0,88
TRC3_PLI	Mardin, Batman, Şirnak, Siirt	91,88	95,75	93,81	1,18

PCGDP data is mostly in TR10 Istanbul region, followed by TR51 Ankara and TR42 Kocaeli, Sakarya, Düzce, Bolu, Yalova. The lowest PcGDP value of TRB2 is in Van, Muş, Bitlis, Hakkari regions. GDP per capita varies between 48,217,41 TL and 12558,19 TL (Table 4).

**Table 4:** GDP per capita levels for statistical regions for 2008–2021.

	Cities	Minimum	Maximum	Mean	Std. deviation
TR10_PcGDP	İstanbul	14,795	140,698	48,217,41	33,956,25
TR51_PcGDP	Ankara	12,579	116,933	40,749,9	27,872,38
TR42_PcGDP	Kocaeli, Sakarya, Düzce, Bolu, Yalova	11,052	118,084	37,857,73	27,871,29
TR21_PcGDP	Tekirdağ, Edirne, Kirklareli	10,843	111,673	34,896,34	26,368,18
TR31_PcGDP	İzmir	10,358	104,791	34,075,48	24,940,25
TR41_PcGDP	Bursa, Eskişehir, Bilecik	10,205	95,985	32,952,14	22,840,34
TR61_PcGDP	Antalya, Isparta, Burdur	10,092	76,977	29,634,59	18,197,14
TR22_PcGDP	Balikesir, Çanakkale	7916	77,553	26,400,9	18,873,59
TR32_PcGDP	Aydin, Denizli, Muğla	8477	72,686	25,548,88	17,429,44
TR33_PcGDP	Manisa, Afyon, Kütahya, Uşak	6656	73,092	24,342,46	18,028,52
TR52_PcGDP	Konya, Karaman	6329	66,393	22,803,69	16,659,07
TR62_PcGDP	Adana, Mersin	6603	67,963	22,256,84	16,337,88
TR72_PcGDP	Kayseri, Sivas, Yozgat	6489	65,159	22,251,44	15,824,37
TR81_PcGDP	Zonguldak, Karabük, Bartin	5496	67,327	21,319,03	16,204,19
TR82_PcGDP	Kastamonu, Çankiri, Sinop	6533	62,758	21,263,38	15,037,18
TR71_PcGDP	Kirikkale, Aksaray, Niğde, Nevşehir, Kirşehir	5743	59,201	20,295,52	14,645,82
TR90_PcGDP	Trabzon, Ordu, Giresun, Rize, Artvin, Gümüşhane	5593	52,417	19,430,26	13,099,33
TRA1_PcGDP	Erzurum, Erzincan, Bayburt	5075	54,845	18,903,5	13,741,42
TRC1_PcGDP	Gaziantep, Adiyaman, Kilis	5084	61,828	18,739,58	15,180,35
TR83_PcGDP	Samsun, Tokat, Çorum, Amasya	5427	50,462	18,452,6	12,456,94
TR63_PcGDP	Hatay, K.Maraş, Osmaniye	5248	57,720	18,258,3	13,816,61
TRB1_PcGDP	Malatya, Elaziğ, Bingöl, Tunceli	4781	51,184	17,432,98	12,735,01
TRC3_PcGDP	Mardin, Batman, Şirnak, Siirt	3640	43,688	14,224,87	10,954,09
TRA2_PcGDP	Ağri, Kars, Iğdir, Ardahan	3327	36,666	12,558,19	9394,76
TRC2_PcGDP	Şanliurfa, Diyarbakir	4084	30,661	11,754,39	7409,83
TRB2_PcGDP	Van, Muş, Bitlis, Hakkari	3061	32,397	11,453,56	8329,64

Number of emergency stations were significantly correlated with TR31 (*r* = 0.903; *P* < .01), TR32 (*r* = 0.771; *P* < .01), TR42 (*r* = −0.798; *P* < .01), TR62 (*r* = 0.837; *P* < .01), TR63 (*r* = −0.749; *P* < .01), TR72 (*r* = −0.719; *P* < .01), TR83 (*r* = 0.873; *P* < .01), TRA2 (*r* = −0.873; *P* < .01), TRB2 (*r* = −0.736; *P* < .01), TRC2 (*r* = 0.697; *P* < .01), and TRC3 (*r* = 0.574; *P* < .01). In total, 11 of 26 were significantly correlated with inequalities (Table 5).

**Table 5:** Spearman’s rho correlation analysis results between price level index of statistical regions with number of emergency services and population per emergency stations.

	Cities	Price level index	
		Number of emergency stations	Population per emergency stations
TR10	İstanbul	0.270	−0.262
TR21	Tekirdağ, Edirne, Kirklareli	−0.073	0.055
TR22	Balikesir, Çanakkale	−0.499	0.490
TR31	İzmir	**0.903**†	**−0.890**†
TR32	Aydin, Denizli, Muğla	**0.771**†	**−0.780**†
TR33	Manisa, Afyon, Kütahya, Uşak	−0.332	0.323
TR41	Bursa, Eskişehir, Bilecik	0.033	−0.046
TR42	Kocaeli, Sakarya, Düzce, Bolu, Yalova	**−0.798**†	**0.793**†
TR51	Ankara	−0.332	0.349
TR52	Konya, Karaman	0.464	−0.477
TR61	Antalya, Isparta, Burdur	−0.446	0.451
TR62	Adana, Mersin	**0.837**†	**−0.824**†
TR63	Hatay, K.Maraş, Osmaniye	**−0.749**†	**0.771**†
TR71	Kirikkale, Aksaray, Niğde, Nevşehir, Kirşehir	−0.371	0.398
TR72	Kayseri, Sivas, Yozgat	**−0.719**†	**0.741**†
TR81	Zonguldak, Karabük, Bartin	0.138	−0.174
TR82	Kastamonu, Çankiri, Sinop	0.051	−0.064
TR83	Samsun, Tokat, Çorum, Amasya	**0.873**†	**−0.881**†
TR90	Trabzon, Ordu, Giresun, Rize, Artvin, Gümüşhane	−0.037	0.051
TRA1	Erzurum, Erzincan, Bayburt	0.231	−0.240
TRA2	Ağri, Kars, Iğdir, Ardahan	**−0.873**†	**0.855**†
TRB1	Malatya, Elaziğ, Bingöl, Tunceli	−0.152	0.165
TRB2	Van, Muş, Bitlis, Hakkari	**−0.736**†	**0.767**†
TRC1	Gaziantep, Adiyaman, Kilis	0.257	−0.248
TRC2	Şanliurfa, Diyarbakir	**0.697**†	**−0.684**†
TRC3	Mardin, Batman, Şirnak, Siirt	0.574*	−0.565*

Bold values are significant differences.

## 4. Discussion

Unlike other health services, health services provided in emergency services stand out more as a public and human right besides human rights. The health services provided in the emergency departments may include serious processes that limit the vital activities of individuals and even result in death in some cases.^[17–19]^ For this reason, access to emergency services is of vital importance and is an individual and social issue that should not be compromised.

According to the changes in the number of emergency services in our country since 2002, there has been a great increase from 481 to 3170. The change in the rate of population per emergency service has been examined to see whether the increase in this number is only numerical or whether there has been a development in terms of content. The same development has been experienced here, and the population per emergency service, which was 138050 in 2002, decreased to 26713 in 2021. In fact, even this shows that since 2002, great developments and steps have been taken regarding the emergency service. However, no sufficient statistical explanation, examination or study has been found on how and how these are distributed by regions. For this reason, the relationship between purchasing power and income per capita variables according to statistical regions, and the number of emergency services and population per emergency department variables were examined in the study.

The price level index, PLI, was used as an indicator of purchasing power.^[20–22]^ According to the variation of the PLI value of the regions in the country, the index is higher in the big cities such as Istanbul, Izmir and Ankara, and it is lower in the eastern provinces. However, the difference between the statistical region with the highest PLI value and the highest statistical region is around 21%, and it is not possible to talk about a quarterly difference of 25%. This indicates that although the inequalities between regional purchasing power and price indices are statistically significant, there are not extreme differences. In terms of price, the differences between the statistical groups of Turkstat are not very extreme in terms of statistics. In addition, there is a linearization deviations in statistical arrangements.^[23]^

Since it would be incomplete and wrong to show purchasing power with the price index alone, per capita income should also be taken into account in order to fully reveal the regional inequalities. Although national income increases in Turkey and countries with similar economic systems, serious differences may arise in terms of purchasing power and its reflections on individuals. Particularly, the fact that developing countries open more to foreign markets and focus on direct sales indicates a structure in which the rich get richer and the gap between the rich and the poor increases. However, although these differences in health services cause injustice, these differences in access to emergency services bring along vital problems.

The results of the correlation analysis reveal that the success achieved with the emergency service and health reform movements by population since 2002 in Turkey has a significant effect for 11 out of 26 statistical regions. In 15 statistical regions, the relationships between the change in the income per capita or the price index of individuals and the number of emergency services and the population per emergency service are not significant. In the health services, which are the most important indicator of the increasing welfare level, it is seen that these developments are not reflected to the individuals sufficiently in 15 statistical regions. In order for the well-intentioned and successful achievements in this regard to yield more effective results, the relationship between regional inequalities and the effectiveness of emergency services should be the subject of more studies and should be followed more by public authorities.

The most important limitation of the research is the difficulties in accessing the statistical data of the distribution of the emergency services regionally and the inability to compile the regional differences with sufficient statistical data. Another important limitation of the study is that the indexes revealing the inequality of regions and statistical region limitations are not adequately presented and the method by which the distinction is made.

The most important contribution of the research to the literature is that it is both a pioneer in the field and a guide in the literature in terms of spreading the increase in the number and capacity of emergency medicine services, which is a success achieved. Emergency health services are not selective or externally decided health services of individuals, they have vital importance. For this reason, it is useful to evaluate and deal with economic and social inequalities within this framework. In this respect, research is important in terms of effective use of public resources and aiming to increase efficiency in health services.

## 5. Conclusion

The results obtained in the research show that the increases in both quantity and capacity and quality of emergency services do not sufficiently take into account regional inequalities. In 15 out of 26 statistical regions, the distribution of the number of emergency services and the ratio to the population is not in a significant relationship. This shows that regional inequalities continue in emergency services. In a more effective health service delivery, it is beneficial to manage emergency services in a way that takes into account not only population density, but also regional inequalities and the frequency of use of health services.

## Acknowledgments

We thank Kadir Yilmaz with his valuable statistics support.

## Author contributions

**Conceptualization:** Erkan Boğa.

**Data curation:** Erkan Boğa.

**Formal analysis:** Erkan Boğa.

**Funding acquisition:** Erkan Boğa.

**Investigation:** Erkan Boğa.

**Methodology:** Erkan Boğa.

**Validation:** Erkan Boğa.

**Writing – original draft:** Erkan Boğa.

**Writing – review & editing:** Erkan Boğa.
